# Blood pressure reduction and anti-inflammatory macrophage augmentation attenuate uterine immune dysregulation and inflammation in mice with salt-sensitive hypertension

**DOI:** 10.1042/CS20255879

**Published:** 2025-12-10

**Authors:** Shobana Navaneethabalakrishnan, Bethany L. Goodlett, Hannah L. Smith, Alyssa Cardenas, Robert A. Montalvo II, Gabriella C. Peterson, Brett M. Mitchell

**Affiliations:** 1 Department of Medical Physiology, Texas A&M College of Medicine, Bryan, Texas, 77807, U.S.A.

**Keywords:** hormone receptors, hypertension, lymphatics, macrophages, uterus

## Abstract

Salt-sensitive hypertension (SSHTN) promotes systemic inflammation, pro-inflammatory immune cell infiltration, and end-organ damage, including in the kidneys and gonads. However, its impact on uterine immune cell populations remains unclear. We hypothesized that SSHTN alters immune cell homeostasis, induces inflammation, and promotes lymphangiogenesis in the uterus, and that these effects can be mitigated by pharmacological blood pressure (BP) reduction and anti-inflammatory macrophage augmentation. To test the hypothesis, female C57BL6/J mice were given nitro-L-arginine methyl ester hydrochloride (0.5 mg/ml) in drinking water for 2 weeks, followed by a 2-week washout period. Mice were then subjected to a 4% high-salt diet (SSHTN) for 3 weeks. Another group of mice received either hydralazine (HYD; 250 mg/l in drinking water), a vasodilator (SSHTN+HYD), or AVE0991 (AVE; 0.58 µmol/kg body weight/day), a nonpeptide Mas receptor agonist, through daily intraperitoneal injections (SSHTN+AVE). Control mice received tap water and a standard diet for the entire treatment period. Flow cytometry data revealed a significant decrease in total uterine CD45+ immune cells, along with an increase in tissue macrophages, in all SSHTN groups compared with the control group. SSHTN mice had increased uterine pro-inflammatory macrophages, dendritic cells, natural killer cells, and CD4+ pro-inflammatory T cells, all of which were mitigated by HYD and AVE treatments. SSHTN promoted uterine inflammation, lymphatic vessel expansion, and altered hormone receptor expression, which were mitigated by pharmacological intervention, highlighting their therapeutic potential in preserving uterine homeostasis and improving reproductive health in women with SSHTN.

## Introduction

Hypertension (HTN), one of the leading causes of cardiovascular diseases and premature death, affects over 1.28 billion adults worldwide [1–4]. Besides cardiovascular and renal morbidity, HTN has been associated with impaired reproductive health [5–7]. Hypertensive females exhibit sexual dysfunction due to altered clitoral and vaginal vasculature, reduced blood flow, and thinning of vaginal and clitoral smooth muscles, resulting in vaginal dryness [8,9]. Additionally, reduced blood flow and nitric oxide levels can lead to fibrosis, decreased lubrication, and dyspareunia. Due to the lower prevalence of HTN in young reproductive women than their male counterparts, there are very limited studies that focus on the mechanisms by which HTN affects female fertility. While most of the existing studies have focused on hypertensive disorders of pregnancy (HDP), there is very limited research investigating the impact of HTN on non-pregnant women. HDP, including preeclampsia and gestational HTN, are well known to cause complications during pregnancy, such as impaired implantation, abnormal placentation, and fetal growth restriction, affecting normal fetal development. Extensive studies have demonstrated that these adverse effects are consequences of impaired vascular remodeling, increased arterial stiffness, and altered matrix metalloproteinase activity that leads to impaired uteroplacental perfusion [10–12]. Studies using both animals and humans have demonstrated a reduction in uterine artery blood flow in hypertensive pregnancies [13,14]. The risks of placental abruption, maternal stroke, renal failure, pulmonary edema, and death are significantly elevated in women with HTN [15–20]. Women with HDP also exhibit increased circulating levels of pro-inflammatory cytokines such as interleukin (IL)-6 and tumor necrosis factor (TNF)-α [21–23]. These findings highlight the impact of HTN-induced inflammation and vascular dysfunction during pregnancy and raise the critical question of whether uterine tissues are similarly altered in non-pregnant females, predisposing them to reproductive complications.

Although there are numerous factors that contribute to HTN, a significant subset of individuals exhibits a unique sensitivity to dietary salt. This results in salt-sensitive HTN (SSHTN), which affects over 50% of the adult population with HTN [4]. Numerous studies in humans and animals have emphasized the critical role of innate and adaptive immune cells in the pathogenesis of SSHTN and end-organ damage [24–26]. Studies have well established that high salt intake directly influences immune cell function, triggering a pro-inflammatory response by simultaneously activating pro-inflammatory macrophages and inhibiting anti-inflammatory macrophages [27–33]. Additionally, isolevuglandin adducts and IL-1β production are elevated during high sodium influx into dendritic cells (DCs) via amiloride-sensitive channels, further promoting the production of IL-17A and interferon (IFN)-γ by T cells [32]. Elevated sodium levels also disrupt regulatory T cell (Treg) function while promoting pro-inflammatory T cell responses, highlighting the complex relationship between salt, blood pressure (BP), and inflammation in HTN [34].

Though the effects of HTN on reproductive health have long been recognized, very little is known about how the disease directly affects uterine health in non-pregnant women [5–7]. Recent research demonstrated a positive correlation between HTN and uterine pathologies, such as fibroid growth and poor vascularization [35–37]. Furthermore, a longitudinal investigation reported that antihypertensive drug treatment was associated with fewer uterine fibroids, whereas untreated or new onset HTN patients displayed increased fibroid occurrences [38]. Moreover, HTN has been associated with irregular menstrual cycles and early menopause [39,40]. Despite these findings, the underlying mechanisms causing these adverse effects, particularly in SSHTN, remain poorly understood. This concerning gap in knowledge highlights the need for further investigation on the impact of SSHTN on uterine tissue and female reproductive health, especially given the increased prevalence of SSHTN in women than men, regardless of ethnicity [41]. Our laboratory and others have established that SSHTN promotes pro-inflammatory skewing of renal and gonadal macrophages and is associated with increased inflammation, inflammation-associated lymphangiogenesis, organ dysfunction, and end-organ damage [7,31,32,42–46]. AVE0991 (AVE), a non-peptide Mas receptor agonist, promotes macrophage polarization toward the anti-inflammatory M2 phenotype by selectively activating the Ang-[1–7]/Mas axis. This pathway modulates key signaling cascades, including suppression of NF-κB and MAPK activity and activation of the JNK/FoxO1 pathway, contributing to its anti-inflammatory effects [47–49]. Recent findings from our group also demonstrated that reduction of BP by use of the vasodilator hydralazine (HYD) and induction of anti-inflammatory macrophages by use of the Mas receptor agonist AVE effectively alleviated end-organ damage in renal and gonadal tissues of mice with SSHTN [45,46]. However, the direct impact of SSHTN on uterine tissue remains largely unknown. It is also unclear whether any observed uterine inflammation and damage in SSHTN is driven primarily by elevated BP, by increased pro-inflammatory macrophage activity, or by a combination of both factors. To address this gap, we utilized two mechanistically distinct interventions: HYD, which lowers systemic BP, and AVE, a Mas receptor agonist that promotes macrophage polarization toward an anti-inflammatory (M2) phenotype. This approach enabled us to dissect the relative contributions of BP reduction and macrophage modulation in mitigating SSHTN-induced uterine inflammation and tissue damage.

Based on the limited research that is currently available, we hypothesized that SSHTN promotes uterine damage by inducing pro-inflammatory skewing of macrophages and altering hormone receptor expression. Additionally, we propose that uterine damage can be mitigated by lowering BP and modulating macrophage polarization toward an anti-inflammatory phenotype, offering potential therapeutic approaches to improve reproductive health under hypertensive conditions.

## Methods

### Animal models

The experimental procedures involving animals were approved by the Texas A&M University Institutional Animal Care and Use Committee (IACUC # 2022-0083) and adhered to the guidelines outlined in the National Institutes of Health (NIH) Guide for the Care and Use of Laboratory Animals. All animal studies were performed at Texas A&M University College of Medicine in Bryan, TX. For this study, wildtype female C57BL/6 J mice, aged 8–10 weeks, were sourced from Jackson Laboratories (Bar Harbor, ME) and allowed a 2-week acclimatization period. To induce SSHTN, female mice were administered nitro-L-arginine methyl ester hydrochloride (0.5 mg/ml; Sigma, St. Louis, MO) in their drinking water for 2 weeks, followed by a 2-week washout period. Afterward, the mice were switched to a 4% high-salt diet (Teklad Envigo, Huntingdon, United Kingdom) for 3 weeks [29,35]. In addition to the high-salt diet, one group of mice was given HYD (250 mg/l; Sigma) in their drinking water to evaluate the effects of pharmacological BP reduction on uterine tissue (SSHTN + HYD). Another group of mice was administered AVE (0.58 μmol/kg body weight/day, Sigma) through daily intraperitoneal injections during the high-salt diet phase (SSHTN + AVE). Mice in the control group received a standard chow diet and tap water. All water and diets were provided *ad libitum*. In total, each group in the current study comprises the following number of female mice: 13 in the control group, 13 in the SSHTN group, 5 in the SSHTN + HYD group, and 8 in the SSHTN + AVE group. At the end of the seventh week, all mice were humanely killed by exsanguination under 5% inhaled isoflurane anesthesia, with death confirmed by cervical dislocation.

### BP measurements

Systolic BP (SBP) was measured weekly using a non-invasive tail cuff method, employing the IITC Life Science BP acquisition system (IITC Inc., Woodland Hills, CA). Prior to measurements, mice were acclimatized for 30 minutes in a quiet environment. They were then transferred to pre-warmed restrainers and placed in a warming chamber set at 34°C. Mice were acclimatized for an additional 5–10 minutes after placement in the restrainers and tail cuffs before SBP readings were taken. Two independent, blinded investigators evaluated the BP tracings to determine SBP values.

### Flow cytometry

Uterine tissues were carefully minced and placed into digestion buffer containing collagenase II (1 mg/ml; Worthington Biochemicals, Lakewood, NJ), DNase I (0.15 mg/ml; Sigma), and dispase II (1 mg/ml; Sigma). The tissue was incubated at 37°C for 30 minutes with continuous agitation using a gentleMACS Octo Dissociator with heaters (Miltenyi Biotec, Bergisch Gladbach, Germany). After digestion, the single-cell suspension was passed through sterile 100 μm and 40 μm strainers, followed by rinsing with Dulbecco’s phosphate-buffered saline (DPBS; Thermo Fisher Scientific, Waltham, MA). Red blood cells were lysed using ACK lysing buffer (Thermo Fisher Scientific), and the cells were washed twice with DPBS before plating. The cell suspension was divided into three equal volumes, and each was assigned to one of three antibody panels, as described in previous studies [45]. Cells were then incubated with one of the following viability dyes: Ghost Dye Violet 510 (Tonbo Biosciences, San Diego, CA), Ghost Dye Red 710 (Tonbo Biosciences), or the Zombie Red Fixable Viability Kit (BioLegend, Inc., San Diego, CA), depending on the antibody panel, for 30 minutes on ice. After a wash with DPBS, the cells were resuspended in a 0.1% fetal bovine serum (FBS) solution and incubated with anti-mouse CD16/CD32 antibody (BD Biosciences, San Jose, CA) for 10 minutes on ice to block non-specific Fc receptor binding. Subsequently, cells were stained with fluorescently conjugated antibodies specific to cell markers, including CD45, CD11b, CD11c, F4/80, CD206, CD3e, CD161, CD4, and CD25, at a 1:100 dilution. Cells stained with CD4 were permeabilized and fixed using the FoxP3 Transcription Factor Staining Buffer Set (Invitrogen, Waltham, MA), followed by intracellular staining with antibodies against IFNg, IL-4, FoxP3, TNFα, and IL-17 for 30 minutes on ice using the same antibody dilutions (1:100). After staining, cells were washed, resuspended in 0.1% FBS solution, and passed through sterile 35 μm strainers. Data were acquired using a BD LSR Fortessa X-20 flow cytometer with FACS DIVA software v9.0 (BD Biosciences) and analyzed using FlowJo v10.8 (FlowJo, LLC, Ashland, OR). A maximum of 500,000 cells was analyzed per sample. Results are presented as percentages of the respective parent populations. A list of antibodies and gating strategies is provided in detail in Supplementary Table S1 and Supplementary Figures S1-S3, respectively.

### Real-time quantitative PCR

Extraction of total RNA from uterine tissues was conducted using a Quick-RNA Miniprep Kit (Zymo Research, Irvine, CA), following the manufacturer’s instructions. cDNA synthesis was done by reverse transcribing 1 µg RNA using an RT^2^ First Strand Kit (Qiagen, Germantown, MD), following the manufacturer’s protocol. Reactions of 10 µl volume were prepared by mixing PowerUp SYBR Green Master Mix (Applied Biosystems, Waltham, MA), nuclease-free water (Invitrogen), and gene primers (10 μM; Sigma), along with cDNA from the uterine tissue. Expression of mRNA was determined by real-time quantitative PCR (qRT-PCR) using a QuantStudio6 Flex Real-Time PCR system with QuantStudio Real-Time PCR Software v1.7.2 (Applied Biosystems). Fold changes of gene expression were calculated using the 2^-ΔΔCT^ method and ubiquitin as an endogenous control. All primers were designed using the National Center for Biotechnology Information (NCBI) Gene Database, reported previously [45], and are listed in Online Supplementary Table S2.

## Immunofluorescence

Uteri were harvested and cross-sectioned, then fixed in 4% paraformaldehyde (Sigma) at 4°C for 24 hours. After fixation, tissues were rinsed with DPBS and embedded in paraffin. The embedded tissues were sectioned into 5 μm slices, which were then deparaffinized, rehydrated, and permeabilized using a 0.1% Triton X-100 solution (Bio-Rad Laboratories, Inc., Hercules, CA). To block non-specific binding, sections were incubated with 10% AquaBlock (EastCoastBio, North Berwick, ME) for 1 hour at room temperature. Next, sections were incubated with primary antibodies: lymphatic vessel endothelial hyaluronan receptor 1 (LYVE1, goat polyclonal; R&D Systems, Minneapolis, MN) for lymphatic endothelial cells and αSMA (rabbit polyclonal; Abcam, Cambridge, UK) for smooth muscle cells, overnight at 4°C. After washing, sections were incubated with Alexa Fluor 488 and Alexa Fluor 594 secondary antibodies (Invitrogen) for 1 hour at room temperature. Following antibody incubation, tissues were mounted with Prolong Gold antifade reagent containing DAPI (Invitrogen). Images were acquired using an Olympus BX51 fluorescence microscope with a DP72 camera (Olympus, Shinjuku, Tokyo, Japan) and were captured at 4× magnification using cellSens Standard software v1.9. For quantification of LYVE1+ lymphatic vessels, images of the entire tissue were obtained at 4× magnification by blinded investigators. The total number of LYVE1+ pixels per image was determined using ImageJ software (v1.54; NIH, Rockville, MD) by setting a threshold to identify positive endothelial staining.

## Statistical analysis

Statistical analyses were performed using GraphPad Prism v8.4.3 (GraphPad Software, Inc., Boston, MA). Results are depicted in dot plots or bar graphs showing mean ± standard error of the mean (SEM). Differences between groups were assessed using one-way analysis of variance (ANOVA), followed by a Tukey post hoc analysis, and statistical significance was considered at *P*<0.05.

## Results

### Treatment with HYD or AVE effectively mitigated uterine immune cells and inflammation in female SSHTN mice

**Figure 1:**
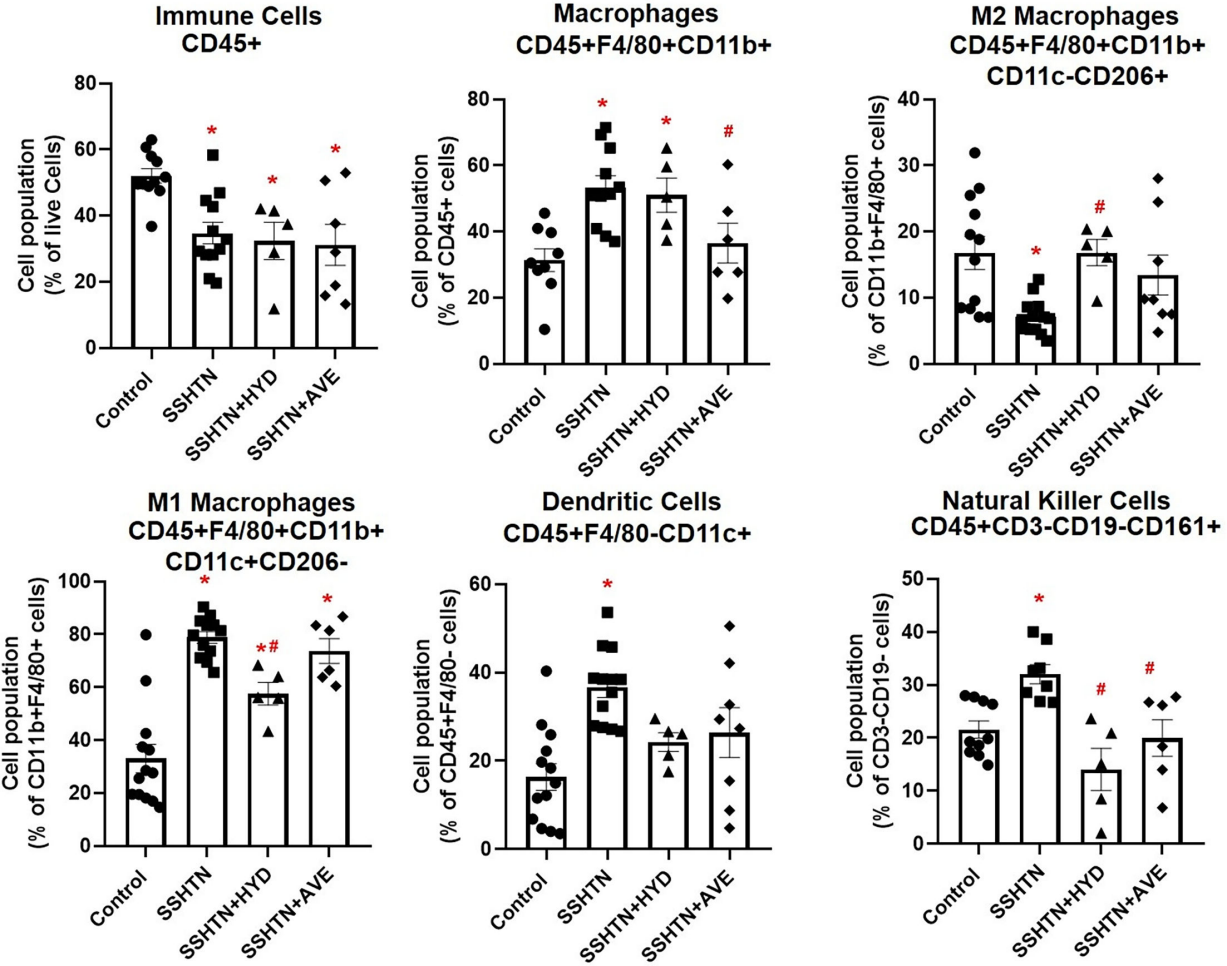
Hydralazine or AVE0991 treatment altered uterine innate immune cell populations. Flow cytometric data examining uterine populations of live immune cells, macrophages, including M2 and M1 macrophage subsets, dendritic cells, and natural killer cells in untreated control, SSHTN, SSHTN + HYD, and SSHTN + AVE female mice. All cell populations are expressed as percentages of their respective parent populations. Results are presented as mean ± SEM, with individual values represented by dots. Statistical analyses were performed with one-way ANOVAs (*n*=5–12 per group). *P<0.05 vs control mice and #*P*<0.05 vs SSHTN mice.

To assess the impact of pharmacological BP reduction and anti-inflammatory macrophage augmentation on uterine immune cell populations, female SSHTN mice were treated with either HYD or AVE, along with a high-salt diet during the final 3 weeks of the protocol. As described in previous studies, SBP was significantly increased in all hypertensive groups (SSHTN, SSHTN + HYD, and SSHTN + AVE) compared with the control group throughout the treatment period (Supplementary Figure S4) [45,46]. However, treatment with HYD or AVE effectively decreased SBP each week during the high-salt diet phase when compared with untreated SSHTN mice (Supplementary Figure S4) [45,46]. The primary objective of the current study is to investigate how SSHTN alters uterine immune cell populations and to determine whether manipulating BP and macrophage polarization can mitigate these changes. To assess these effects, flow cytometric analysis was performed, revealing a significant decrease in total CD45+ immune cells in the uteri of SSHTN mice, regardless of whether they received either intervention (Figure 1). Uterine CD11b + F4/80 + macrophages were increased significantly in the SSHTN and SSHTN + HYD groups compared with the control group, whereas AVE treatment prevented this increase and significantly decreased these macrophages in the SSHTN + AVE group when compared with the SSHTN group (Figure 1). Anti-inflammatory M2 macrophage populations were significantly decreased in the uteri of SSHTN mice compared with control uteri (Figure 1). Both HYD and AVE treatments prevented this decrease in their respective mouse groups, and SSHTN + HYD mice showed an increase in uterine M2 macrophages compared with untreated SSHTN mice (Figure 1). We observed a significant increase in pro-inflammatory M1 macrophages in the uteri of all hypertensive mice compared with control uteri; however, SSHTN + HYD mice had decreased uterine M1 macrophages compared with SSHTN mice (Figure 1). In SSHTN mice, uterine DCs were increased significantly, and both types of treatment attenuated this increase (Figure 1). Additionally, there was a significant increase in natural killer (NK) cells in the uteri of SSHTN mice compared with control uteri (Figure 1). Treatment with HYD and AVE both significantly decreased NK cell populations in the uteri of SSHTN mice (Figure 1).

**Figure 2:**
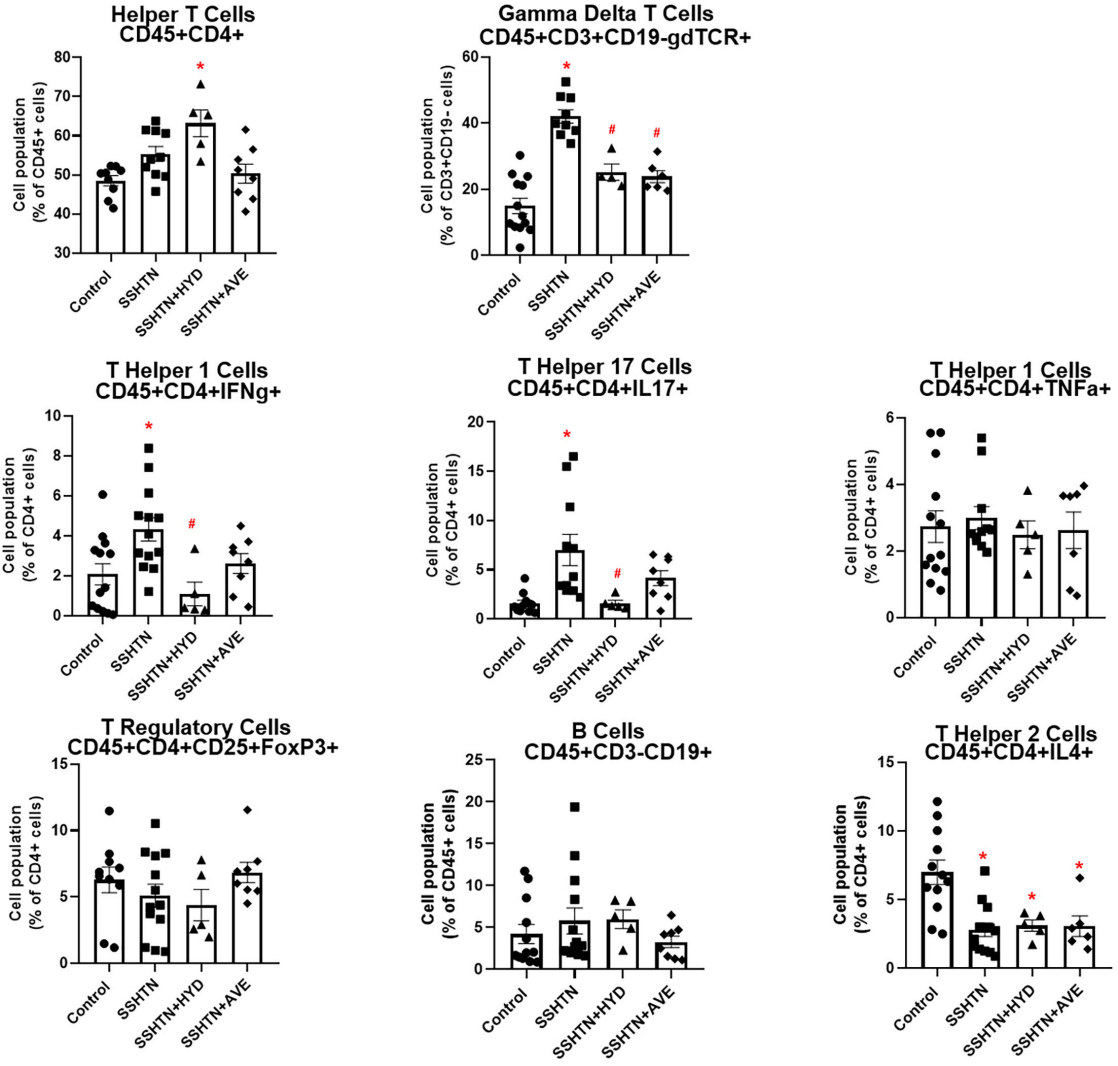
Hydralazine or AVE0991 treatment altered uterine adaptive immune cell populations. Flow cytometric data examining uterine populations of helper T cells, gamma delta T cells, T helper 1 cells, T helper 17 cells, T regulatory cells, B cells, and T helper 2 cells in untreated control, SSHTN, SSHTN + HYD, and SSHTN + AVE female mice. All cell populations are expressed as percentages of their respective parent populations. Results are presented as mean ± SEM, with individual values represented by dots. Statistical analyses were performed with one-way ANOVAs (*n*=4–12 per group). *P<0.05 vs control mice and #*P*<0.05 vs SSHTN mice.

Next, we examined the impact of SSHTN, HYD, and AVE on adaptive immune cell populations within the uterus and found a significant increase in uterine CD4+ T cells in the SSHTN + HYD group, while no changes were observed in the other groups (Figure 2). Additionally, there was a significant increase in uterine gamma delta T cells in SSHTN mice, which was mitigated by treatment with HYD or AVE (Figure 2). Further analysis revealed that CD4+IFNg+ T helper 1 (Th1) and CD4+IL17+ T helper 17 (Th17) cells were increased significantly in the uteri of SSHTN mice compared with control uteri (Figure 2). This effect was mitigated in the treated SSHTN groups, with SSHTN + HYD mice showing significantly decreased uterine Th1 and TH17 cells compared with untreated SSHTN mice (Figure 2). CD4+TNFα+ Th1 cells, CD4+CD25+FoxP3+ Tregs, and CD3−CD19+ B cells remained unaltered among all groups (Figure 2). CD4+IL4+ T helper 2 (Th2) cells were significantly decreased in the uteri of all SSHTN mice, regardless of treatment (Figure 2). These findings demonstrate that SSHTN induces alterations in uterine immune cell populations, which can be mitigated by pharmacological intervention with HYD or AVE, suggesting potential therapeutic strategies.

**Figure 3:**
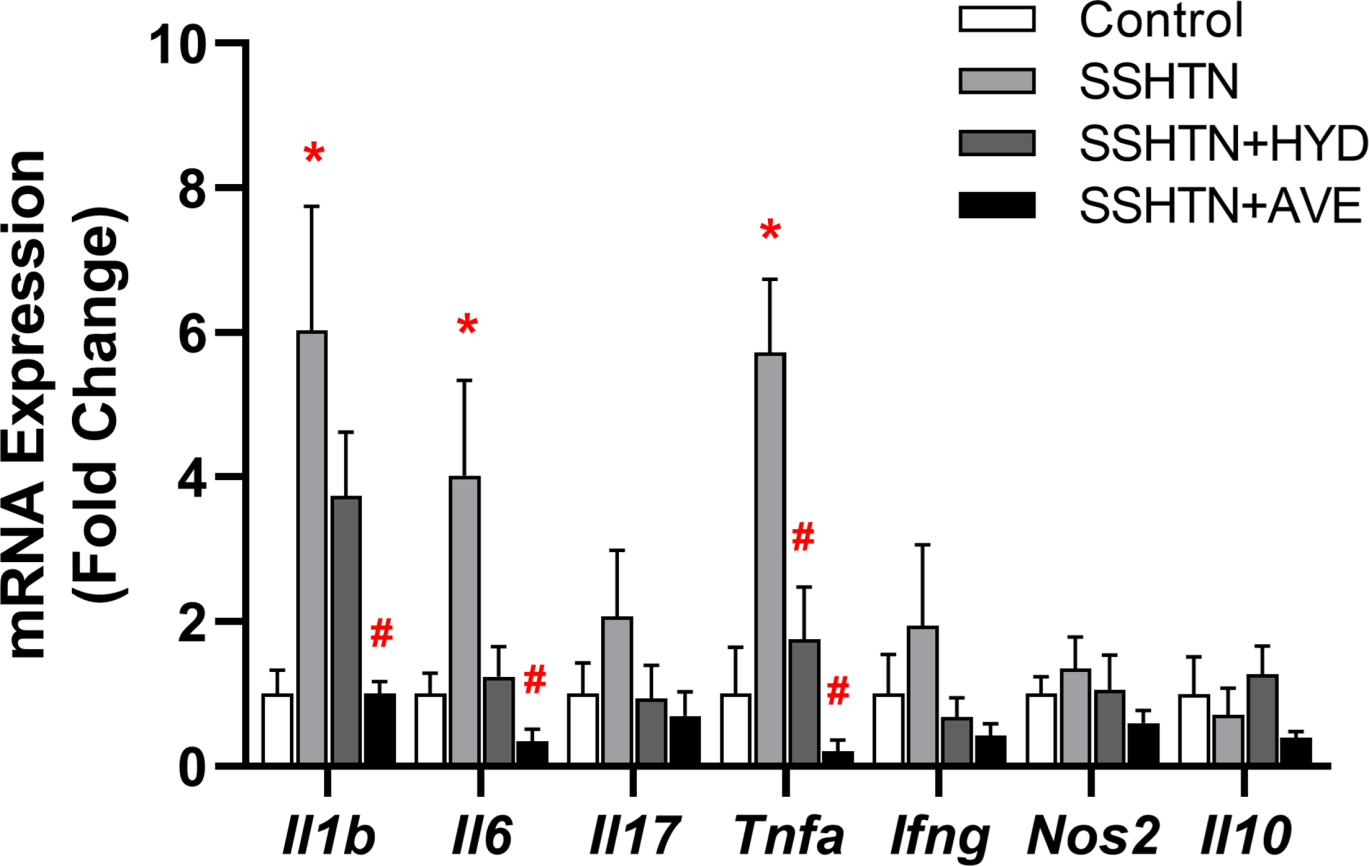
Hydralazine or AVE0991 treatment decreased uterine pro-inflammatory cytokine expression. Uterine cytokine expression changes in untreated control, SSHTN, SSHTN + HDZ, and SSHTN + AVE female mice. Results are presented as mean ± SEM, and statistical analyses were performed with one-way ANOVAs (*n*=3–5 per group). *P<0.05 vs control mice and #*P*<0.05 vs SSHTN mice.

We performed qRT-PCR to determine whether the observed immune cell changes were associated with uterine inflammation. The results revealed a significant increase in the expression of the pro-inflammatory cytokines *Il1b*, *Il6*, and *Tnfα* in the uteri of SSHTN mice, and this was mitigated in both treated SSHTN groups (Figure 3). Uterine *TNFα* expression was decreased significantly in SSHTN + HYD and SSHTN + AVE mice compared with SSHTN mice, while SSHTN + AVE mice showed additional decreases in uterine expression of *Il1b* and *Il6* (Figure 3). Uterine *Il17*, *Ifng*, *Nos2*, and *Il10* expression remained unchanged across all experimental groups (Figure 3).

### Treatment with HYD or AVE effectively mitigated uterine lymphangiogenesis in female SSHTN mice

**Figure 4:**
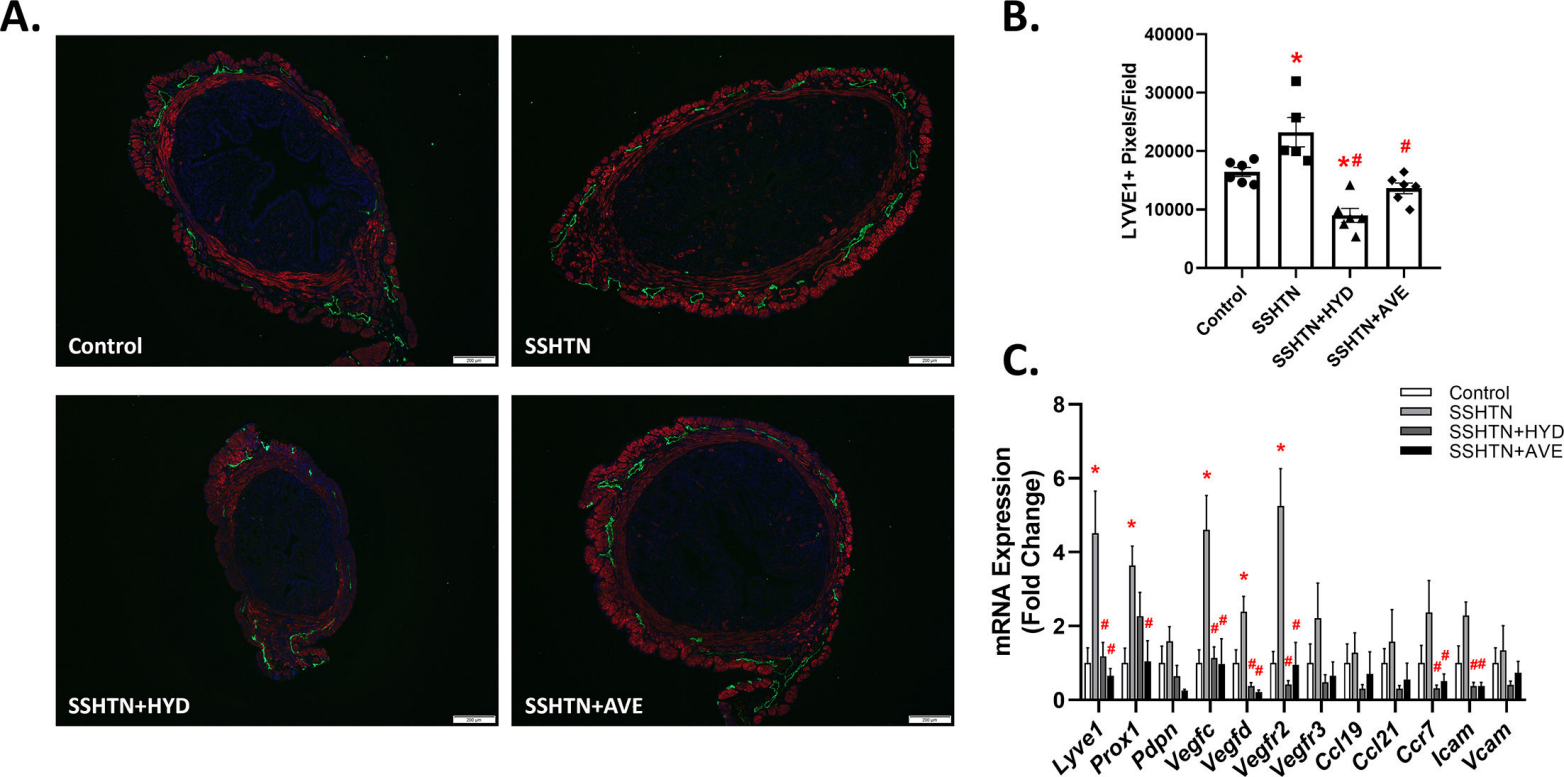
Hydralazine or AVE0991 treatment decreased uterine lymphatic vessel density. (**A**) LYVE1 immunofluorescence in uteri of control, SSHTN, SSHTN + HYD, and SSHTN + AVE female mice. LYVE1 is labelled green, aSMA is labelled red, and DAPI is labelled blue. Images were taken at 4× and scale bars = 200 μm. (**B**) Uterine lymphatic density in control, SSHTN, SSHTN + HYD, and SSHTN + AVE female mice as determined by quantification of LYVE1+ pixels per field (*n*=5–6 per group). (**C**) Uterine expression changes in lymphangiogenesis-related genes in control, SSHTN, SSHTN + HYD, and SSHTN + AVE female mice (*n*=3–5 per group). Results are presented as mean ± SEM, and statistical analyses were performed with one-way ANOVAs. *P<0.05 vs control mice and #*P*<0.05 vs SSHTN mice.

Extensive studies from our laboratory and others have demonstrated that SSHTN promotes inflammation and inflammation-associated lymphangiogenesis in organs such as the kidneys, testes, and ovaries [7,26,27,43–46]. Based on these findings, we investigated whether SSHTN affects uterine lymphatic vessel density in a similar manner. To evaluate lymphatic vessel density, uterine sections were immunolabeled with LYVE1, a marker for lymphatic vessels. Uterine lymphatic density was increased significantly in SSHTN mice compared with controls, as evidenced by quantitative analysis of LYVE1+ pixels per field (Figure 4A and B). Pharmacological intervention with HYD or AVE attenuated the increased lymphatic density in the uteri of their respective groups when compared with untreated SSHTN uteri (Figure 4A and B). Additionally, SSHTN + HYD mice had significantly decreased uterine lymphatic density compared with control mice (Figure 4A and B).

We next evaluated uterine gene expression of key lymphatic vessel markers, chemokines, and adhesion molecules by qRT-PCR and found a significant increase in *Lyve1* and *Prox1* expression in the uteri of SSHTN mice compared with control uteri, which was mitigated by both types of treatment (Figure 4C). SSHTN + HYD and SSHTN + AVE mice had significantly decreased uterine *Lyve1* expression when compared with SSHTN uteri, and SSHTN + AVE mice also had decreased uterine *Prox1* expression (Figure 4C). Notably, uterine expression of *Pdpn* was unaltered across all experimental groups (Figure 4C). Further analysis revealed a significant increase in the expression of the lymphangiogenic factors vascular endothelial growth factor (*Vegf)c* and *Vegfd*, as well as their receptor, vascular endothelial growth factor receptor (*Vegfr)2*, in the uteri of SSHTN mice compared with control uteri (Figure 4C). Both HYD and AVE treatments significantly decreased uterine expression of these lymphangiogenic genes in their respective mouse groups (Figure 4C). Additionally, SSHTN + HYD and SSHTN + AVE mice demonstrated decreased uterine expression of the chemokine receptor *Ccr7* and the adhesion molecule *Icam* when compared with SSHTN mice (Figure 4C). Expression of *Vegfr3*, *Ccl19*, *Ccl21*, and *Vcam* was not altered significantly in the uteri of all groups (Figure 4C). Overall, these findings suggest that SSHTN promotes an increase in uterine lymphatic vessel density, which can be partially reversed by pharmacological BP reduction and anti-inflammatory macrophage augmentation.

### Treatment with HYD or AVE effectively mitigated uterine hormone expression in female SSHTN mice.

**Figure 5:**
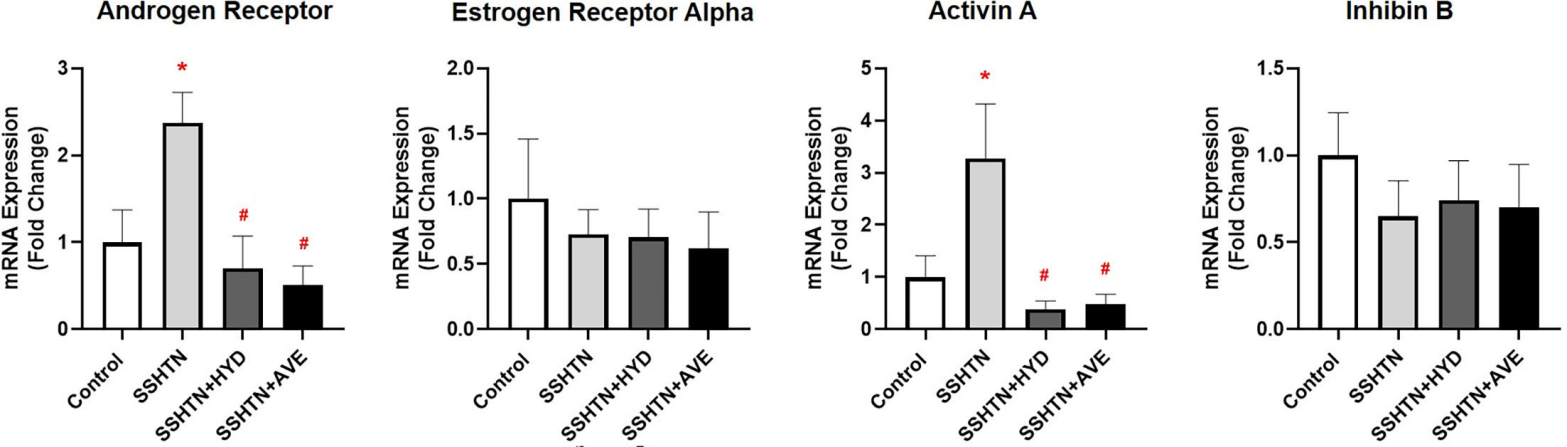
Hydralazine or AVE0991 treatment improved uterine hormone receptor expression in SSHTN. Uterine expression of hormone receptors in untreated control, SSHTN, SSHTN + HDZ, and SSHTN + AVE female mice. Results are presented as mean ± SEM, and statistical analyses were performed with one-way ANOVAs (*n*=4–5 per group). *P<0.05 vs control mice and #*P*<0.05 vs SSHTN mice.

The uteri of SSHTN mice demonstrated a significant increase in the expression of the hormone receptor *Ar* compared with control uteri (Figure 5). Both HYD and AVE treatments led to a significant decrease in uterine *Ar* expression (Figure 5). Similarly, uterine *Inhba* (Activin A) expression was increased in SSHTN mice compared with control mice, while treatments with HYD and AVE significantly decreased uterine *Inhba* expression compared with untreated SSHTN mice (Figure 5). Uterine *Era* and *Inhbb* (Inhibin B) expression remained unaltered in the experimental groups (Figure 5).

## Discussion

SSHTN is known to promote pro-inflammatory macrophage skewing, inflammation, and end-organ damage in renal and gonadal tissues; however, little is known about its direct impact on uterine health [7,31,32,42–46]. Uterine dysfunction, including fibroid production and growth, poor vascularization, irregular menstrual cycles, and early menopause, has been well associated with HTN, yet the underlying mechanisms remain unclear [35–37,39,40]. Given the higher prevalence of SSHTN in women, understanding its effects on uterine immune homeostasis is needed [50]. The current study addresses this gap in knowledge by investigating how SSHTN alters uterine immune cell populations and whether BP reduction and anti-inflammatory macrophage induction can mitigate these changes. We demonstrated that SSHTN induces uterine damage by promoting pro-inflammatory immune cell accumulation, inflammation, lymphangiogenesis, and altering hormone receptor expression in the uterus. In addition, HYD and AVE each attenuated not only SSHTN but also uterine pro-inflammatory immune cells, inflammation, and lymphangiogenesis. These findings present options for targeted interventions addressing both BP and macrophage polarization to maintain uterine health in SSHTN.

As reported in previous studies, SSHTN mice had significantly elevated SBP, and both HYD and AVE treatments effectively lowered SBP during each week of the high-salt diet treatment [45,46]. However, despite reductions in BP, overall uterine immune cell populations (CD45+) in SSHTN mice remained significantly reduced, independent of pharmacological intervention. A key observation was the significant increase in uterine macrophages in SSHTN mice, which persisted with HYD treatment but was significantly attenuated by AVE treatment. Further characterization revealed a pro-inflammatory skewing of uterine macrophages in SSHTN mice, marked by an increase in pro-inflammatory M1 macrophages and a simultaneous decrease in anti-inflammatory M2 macrophages. While reducing M1 macrophages, HYD therapy increased the percentage of uterine M2 macrophages, showcasing its anti-inflammatory properties. On the other hand, uterine M2 macrophages in AVE-treated SSHTN mice were not increased but were protected from the decrease seen in untreated SSHTN. These findings are consistent with previous studies from our laboratory, which reported a similar M1/M2 macrophage imbalance in the kidneys and gonads of SSHTN mice. This was alleviated by lowering BP and inducing anti-inflammatory macrophage populations [45,46]. Furthermore, the observed immune dysregulation in SSHTN mice parallels findings in endometriosis, a chronic inflammatory uterine disorder associated with increased pro-inflammatory macrophages [51–53]. This shift toward a pro-inflammatory immune environment is associated with chronic pain, impaired tissue healing, and infertility [54]. The predominance of uterine M1 macrophages in both SSHTN and endometriosis suggests a common inflammatory mechanism that may underlie reproductive dysfunction. The persistence of immune dysregulation in SSHTN mice despite BP normalization suggests that immune cell activation may also contribute to disruption of uterine immune homeostasis. Notably, AVE treatment demonstrated more efficacy than HYD in attenuating pro-inflammatory immune cells, highlighting its immunomodulatory effects are, at least in part, independent of BP reduction.

Our study reveals that SSHTN not only alters macrophage populations in the uterus but also increases uterine DCs and NK cells, which are both key mediators of inflammation and immune surveillance [55]. Our observation of elevated uterine DCs in SSHTN mice aligns with our previous findings in the kidneys and gonads, suggesting systemic immune activation [45,46]. Notably, both HYD and AVE treatments reduced uterine DC populations, indicating that BP reduction and macrophage modulation can mitigate antigen-presenting cell activation in the uterus. Similarly, uterine NK cells were elevated significantly in SSHTN mice but were reduced by both treatments, reinforcing the role of these cells in inflammatory regulation. Additionally, SSHTN mice exhibited increases in pro-inflammatory Th1 (CD4+IFNγ+) and Th17 cells in the uterus. AVE treatment prevented this increase in uterine Th1 and Th17 cells, while HYD treatment decreased these cell populations. Interestingly, uterine Th2 cells were decreased significantly across all hypertensive groups, regardless of treatment, pointing to a shift toward a pro-inflammatory state in the uterus. These immune alterations are consistent with findings in reproductive disorders like endometriosis and polycystic ovary syndrome, where uterine DCs, macrophages, NK cells, and pro-inflammatory CD4+ cells are increased. This activation leads to increased production of cytokines, including IL-6, IL-8, IL-17, MCP-1, and GM-CSF, thereby exacerbating the inflammatory milieu [52,56–58]. These findings highlight that SSHTN-induced uterine immune dysregulation parallels mechanisms observed in reproductive disorders, emphasizing its potential impact on uterine health. The ability of antihypertensive treatments to modulate immune cell populations suggests a therapeutic avenue for mitigating SSHTN-associated uterine inflammation and dysfunction. Future studies are needed to explore the mechanistic links between HTN, inflammation, and reproductive health to develop targeted interventions.

We observed increased uterine lymphatic vessel density in SSHTN mice, consistent with our previous findings of inflammation-driven lymphangiogenesis in the kidneys and gonads [7,44–46]. This observation further supports previous research demonstrating that macrophage-derived VEGF-C and VEGF-D, through VEGFR signaling, drive pathological lymphangiogenesis in endometrial tissues during endometriosis [59,60]. The observed increase in uterine lymphatic density in SSHTN mice may reflect a similar inflammatory response, with macrophages contributing to the elevated production of pro-inflammatory cytokines and lymphangiogenic factors. Pharmacological intervention with HYD or AVE significantly reduced this increase in lymphatic vessel density, supporting the role of these treatments in mitigating inflammation-driven vascular remodeling. These findings suggest that BP reduction and modulation of macrophage activation can suppress excessive uterine lymphangiogenesis, potentially providing therapeutic strategies for managing inflammation-associated tissue remodeling in the uterus during SSHTN.

In the current study, SSHTN mice exhibited increased *Ar* expression, which was decreased significantly by both HYD and AVE treatments. Dysregulation of *A*r in SSHTN may contribute to uterine dysfunction, like what is observed in women with polycystic ovary syndrome, where elevated serum androgens and endometrial *Ar* expression impair reproductive performance [61]. Supporting this, androgen receptor blockade in pregnant rats alleviated endocrine, metabolic, and fertility impairments caused by dihydrotestosterone and insulin, restoring normal endometrial receptivity and decidualization gene expression [62]. The expression of *Era* remained unchanged in SSHTN mice, suggesting selective disruption of androgen signaling.

Inhibins are heterodimeric glycoproteins composed of a common α-subunit linked to either a βA-subunit (inhibin A) or a βB-subunit (inhibin B) [63]. In contrast, activins are homodimers made of two β-subunits, either βA (activin A) or βB (activin B), or a heterodimer of both βA and βB (activin AB) [63]. Functionally, inhibin B suppresses follicle-stimulating hormone (FSH) synthesis and secretion from the anterior pituitary, while activins exert the opposite effect by stimulating FSH secretion [63]. The balance between these signaling molecules is essential for regulating ovarian folliculogenesis, endometrial function, and overall reproductive homeostasis [63,64]. In the current study, *Inhba* expression was increased significantly in SSHTN mice and returned to baseline levels by HYD and AVE treatments, while *Inhbb* remained unaffected. The elevated expression of *Inhba* may reflect disruptions in the hypothalamic-pituitary-ovarian axis and its influence on uterine function indirectly through hormonal imbalance. Interestingly, pharmacological intervention with HYD and AVE, which reduce BP and augment anti-inflammatory macrophages, respectively, was both associated with a partial restoration of uterine activin A expression. While the exact mechanism remains unclear, we speculate that improved uterine perfusion, reduced oxidative stress, and inflammation might have restored the endocrine responsiveness of the uterine tissue. It is well known that HTN impairs uterine vascular remodeling and endocrine signaling, and reversing these effects may restore hormonal sensitivity and receptor expression [65]. It is important to acknowledge that the current study did not assess circulating levels of key reproductive hormones (e.g. estradiol, androgens, progesterone, FSH, LH, Activin A, and Inhibin B) or nitric oxide (NO) bioavailability. Such measurements would enhance our understanding of the endocrine and vascular alterations occurring in the uterus under SSHTN conditions. Future investigations incorporating comprehensive hormone profiling and NO assessment are warranted to elucidate the mechanistic links between immune, endocrine, and vascular pathways in SSHTN.

Our findings highlight that SSHTN disrupts uterine immune homeostasis by increasing pro-inflammatory immune cell infiltration, skewing macrophage polarization toward an M1 phenotype, and altering uterine hormone receptor expression. While BP reduction provides beneficial immunomodulatory effects, macrophage-targeted therapies, such as AVE, may offer additional benefits by selectively modulating immune cell populations. These results have significant implications for reproductive health in hypertensive women, highlighting the need for targeted interventions beyond BP control to mitigate uterine immune dysfunction. Future studies are needed that investigate the underlying molecular mechanisms linking SSHTN, uterine immune dysregulation, and reproductive health outcomes.

**Clinical Perspectives**

- Salt-sensitive hypertension (SSHTN) disproportionately affects females and is associated with reproductive dysfunction. However, the direct impact of SSHTN on uterine tissue remains largely unknown, and it remains unclear whether blood pressure (BP) reduction and the augmentation of anti-inflammatory macrophages could mitigate uterine tissue damage.
- The data presented demonstrates that both hydralazine (HYD) and AVE0991 (AVE) improve uterine health in SSHTN. Each drug improved the uterine inflammatory environment by decreasing pro-inflammatory immune cell accumulation and increasing anti-inflammatory immune cell populations (HYD) or decreasing pro-inflammatory cytokine expression (AVE). Both drugs also decreased uterine inflammation-associated lymphangiogenesis and improved expression of uterine hormone receptors.
- This study provides further support for the use of HYD and AVE in hypertensive treatment plans to aid in lowering BP and protecting reproductive function in females.

## Supplementary material

online supplementary material 1.

## Data Availability

All supporting data are included within the article or available in previous publications.

## Open Access

This article has been published open access under our Subscribe to Open programme, made possible through the support of our subscribing institutions, learn more here: https://portlandpress.com/pages/open_access_options_and_prices#conditional

## Abbreviations

ACK: Ammonium-Chloride-Potassium
ANOVA: one-way analysis of variance
AVE: AVE0991
Ar: Androgen receptor
BP: blood pressure
Ccl: Chemokine ligand
DAPI: 4′,6-diamidino-2-phenylindole dihydrochloride
DC: dendritic cell
DPBS: Dulbecco’s phosphate-buffered saline
Er: Estrogen receptor
FBS: fetal bovine serum
FSH: follicle-stimulating hormone
FoxO1: Forkhead box O1
GM-CSF: Granulocyte-macrophage colony-stimulating factor
HDP: hypertensive disorders of pregnancy
HTN: hypertension
HYD: hydralazine
IFN: interferon
IL: interleukin
Icam: Intercellular adhesion molecule
Inhba: Inhibin beta-A subunit
Inhbb: Inhibin beta-B subunit
JNK: c-Jun N-terminal Kinase
LYVE1: lymphatic vessel endothelial hyaluronan receptor 1
MAPK: Mitogen-activated protein kinase
MCP-1: Monocyte chemoattractant protein-1
NK: natural killer
NO: nitric oxide
Pdpn: Podoplanin
SBP: systolic blood pressure
SEM: standard error of the mean
SSHTN: salt-sensitive hypertension
TNF: tumor necrosis factor
Th1: T helper 1
Th2: T helper 2
Th17: T helper 17
Tregs: regulatory T cells
VEGF: vascular endothelial growth factor
VEGFR: vascular endothelial growth factor receptor
Vcam: Vascular cell adhesion molecule
qRT-PCR: real-time quantitative PCR
α-SMA: Alpha smooth muscle actin
