# Supplementary material for: Blood pressure reduction and anti-inflammatory macrophage augmentation attenuate uterine immune dysregulation and inflammation in mice with salt-sensitive hypertension
Source: Clin Sci (Lond). 2025 Dec 10;139(23):1629–42. doi: 10.1042/CS20255879 (PMC12751045; doi:10.1042/CS20255879)
Supplement: online supplementary material 1. [file CS-139-23-CS20255879-s001.docx]

**Title**

**Blood Pressure Reduction and Anti-inflammatory Macrophage Augmentation Attenuate Uterine Immune Dysregulation and Inflammation in Mice with Salt-Sensitive Hypertension**

**Authors and Affiliations**

Shobana Navaneethabalakrishnan, Bethany L. Goodlett, Hannah L. Smith, Alyssa Cardenas, Robert A. Montalvo II, Gabriella C. Peterson, Brett M. Mitchell

Department of Medical Physiology, Texas A&M College of Medicine, Bryan, Texas, 77807, USA

**Corresponding Author**

Dr. Brett M. Mitchell, 8447 John Sharp Parkway, Medical Research Education Building II, Bryan, Texas, 77807, USA

Tel: +1 979 436 0751; E-mail: [brettmitchell@tamu.edu](mailto:brettmitchell@tamu.edu). ORCID: 0000-0002-2575-8761

**Supplemental Materials**

**Online Tables**

*Online Table I. Flow cytometry antibody panel descriptions for mouse uterus.*

| **Panel** | **Innate Immune Cells** | | | | | | | | |
| --- | --- | --- | --- | --- | --- | --- | --- | --- | --- |
| **Fluorochrome** | BV421 | FITC | | APC | | PE-Cy7 | BV785 | | Ghost Dye Red 710 |
| **Antigen** | CD45.2 | CD11b | | CD11c | | F4/80 | CD206 | | Live/dead |
| **Final Conc (μg/mL)** | 2 | 5 | | 2 | | | | |  |
| **Dilution Factor** | 1:100 | | | | | | | | 1:400 |
| **Clone** | 104 | M1/70 | | N418 | | BM8 | C068C2 | |  |
| **Manufacturer** | BD | BioLegend | | BioLegend | | BioLegend | BioLegend | | Tonbo |
| **Panel** | **Natural Killer Cells** | | | | | | | | |
| **Fluorochrome** | Pacific Blue | | | BV605 | | | Zombie Red | | |
| **Antigen** | CD3e | | | CD161 | | | Live/dead | | |
| **Final Conc (μg/mL)** | 2 | | | | | |  | | |
| **Dilution Factor** | 1:100 | | | | | | 1:200 | | |
| **Clone** | 500A2 | | | PK136 | | |  | | |
| **Manufacturer** | BD | | | BioLegend | | | BioLegend | | |
| **Panel** | **CD4-Lineage T Cells** | | | | | | | | |
| **Fluorochrome** | BV421 | | PE-Cy5 | | PE-Cy7 | | | Ghost Dye Violet 510 | |
| **Antigen** | CD45.2 | | CD4 | | CD25 | | | Live/dead | |
| **Final Conc (μg/mL)** | 2 | | | | | | |  | |
| **Dilution Factor** | 1:100 | | | | | | | 1:400 | |
| **Clone** | 104 | | RM4-5 | | PC61 | | |  | |
| **Manufacturer** | BD | | BioLegend | | BioLegend | | | Tonbo | |
| **Panel** | **CD4-Lineage T Cells (Intracellular)** | | | | | | | | |
| **Fluorochrome** | BV605 | BV711 | | AlexaFluor 700 | | BV421 | APC-Cy7 | | |
| **Antigen** | IFNg | IL4 | | FoxP3 | | TNFa | IL17a | | |
| **Final Conc (μg/mL)** | 2 | | | | | | | | |
| **Dilution Factor** | 1:100 | | | | | | | | |
| **Clone** | XMG1.2 | 11B11 | | FJK-16s | | MP6-XT22 | TC11-18H10.1 | | |
| **Manufacturer** | BioLegend | BioLegend | | eBioscience | | BD | BioLegend | | |

Abbreviations: APC = allophycocyanin; BV = brilliant violet; FITC = fluorescein isothiocyanate; PE = phycoerythrin; PerCP-Cy5.5 = peridinin chlorophyll protein complex cyanine 5.5

*Online Table II. Primer sequences for qRT-PCR analysis of murine uterus.*

| **Target** | **Forward (5’ to 3’)** | **Reverse (5’ to 3’)** |
| --- | --- | --- |
| *Ar*  *Ccl19*  *Ccl21*  *Ccr7*  *Era*  *Icam*  *Ifng*  *Il1b* | CCCTGAGGCCGCTAACATAG  GGGGTGCTAATGATGCGGAA  CCCTGCTTCAACCATTACATCTGC  TGTACGAGTCGGTGTGCTTC  AATTCTGACAATCGACGCCAG  GTGATGCTCAGGTATCCATCCA  TCAAGTGGCATAGATGTGGAAGAA  GCCACCTTTTGACAGTGATGAG | GGGCTTGAGGAGAACCATCC  CCTTAGTGTGGTGAACACAACA  CCTGCTGTCTCCTTCCTCATTCC  GGTAGGTATCCGTCATGGTCTTG  GTGCTTCAACATTCTCCCTCCTC  CACAGTTCTCAAAGCACAGCG  TGGCTCTGCAGGATTTTCATG  GACAGCCCAGGTCAAAGGTT |
| *Il6*  *Il10* | GAGGATACCACTCCCAACAGACC  GCTCTTACTGACTGGCATGAG | AAGTGCATCATCGTTGTTCATA  CGCAGCTCTAGGAGCATGTG |
| *Pdpn* | ACCGTGCCAGTGTTGTTCTG | AGCACCTGTGGTTGTTATTTTGT |
| *Prox1* | CTCTTGCCTCGCTATCCCC | CACAGTCCCACTGACGTACC |
| *Tnfa* | GAGAAAGTCAACCTCCTCTCTG | GAAGACTCCTCCCAGGTATATG |
| *Ubc*  *Vcam*  *Vegfc*  *Vegfd*  *Vegfr2*  *Vegfr3* | GCCCAGTGTTACCACCAAGAAG  AGTTGGGGATTCGGTTGTTCT  CAGTGTCAGGCAGCTAACAAG  TGGCAAGACTTTTGAGCTTCAA  GCCCTGCTGTGGTCTCACTAC  ATCAGAAGATCGGGCGCTGTTGTA | GCTCTTTTTAGATACTGTGGTGAG  CCCCTCATTCCTTACCACCC  GAAGGTCCACAGACATCATGGAA  AAATCGCGCACTCTGAGGA  CAAAGCATTGCCCATTCGAT  TGTGTCATGTCCGCCCTTCAGTTA |
| All sequences were verified through National Center for Biotechnology Information Primer-BLAST and single products were confirmed with a melting point dissociation step post amplification. | | |
| Ar, androgen receptor; Ccl19, chemokine ligand 19; Ccl21, chemokine ligand 21; Ccr7, C-C chemokine receptor type 7; Cldn11, Era, estrogen receptor alpha; Icam, intercellular adhesion molecule; Ifng, interferon gamma; Il1b, interleukin 1 beta; Il6, interleukin 6; Il10, interleukin 10; Il17, interleukin 17; Inhba, inhibin beta a subunit; Inhbb, inhibin beta b subunit; Lyve1, lymphatic vessel endothelial hyaluronan receptor 1; Nos2, nitric oxide synthase 2; Pdpn, podoplanin; Prox1, prospero homeobox 1; Tnfa, tumor necrosis factor alpha; Ubc, ubiquitin; Vcam, vascular cell adhesion molecule; Vegfc, vascular endothelial growth factor C; Vegfd, vascular endothelial growth factor D; Vegfr2, vascular endothelial growth factor receptor 2; Vegfr3, vascular endothelial growth factor receptor 3. | | |

**Supplementary Figures**


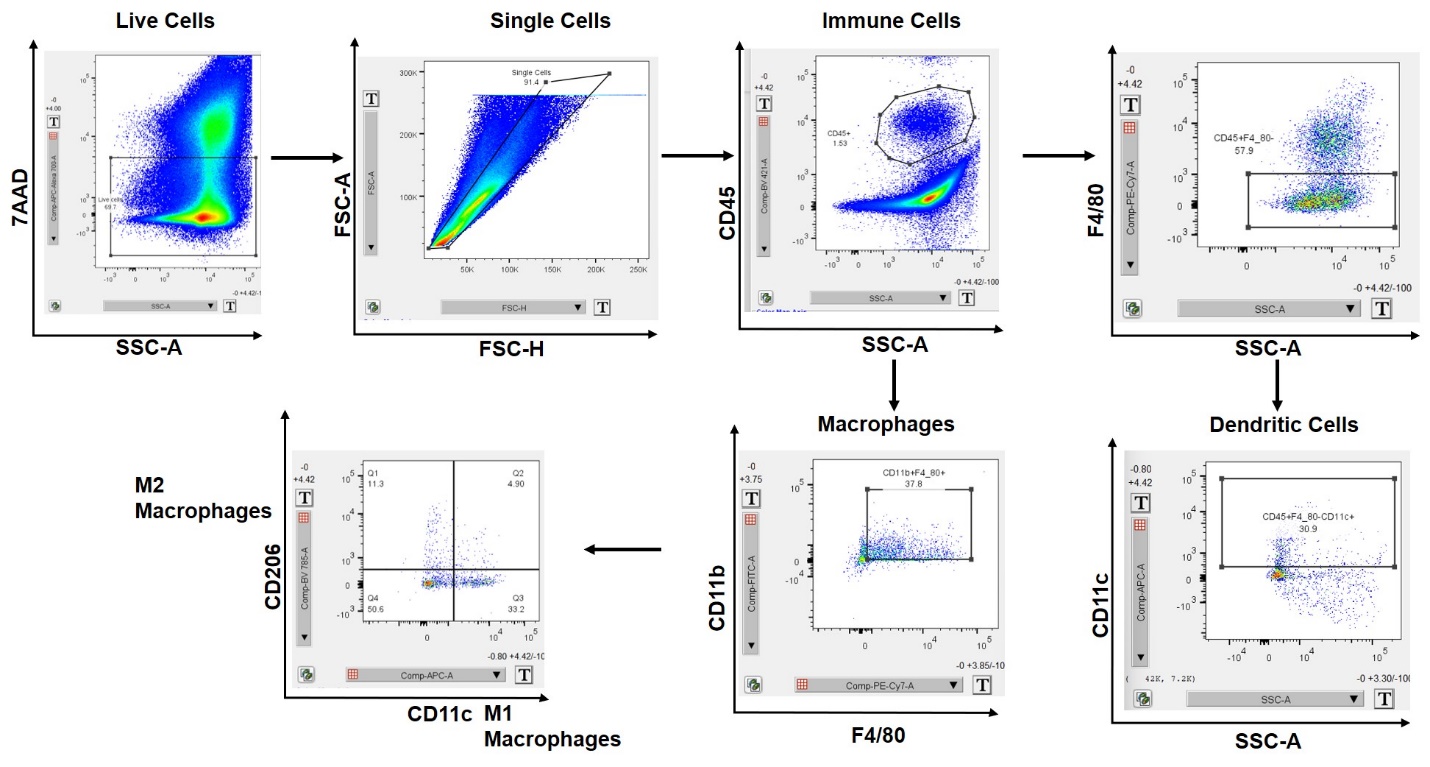


**Supplementary Figure S1.** Gating strategy for innate immune cells in uterus. Uterine single-cell suspensions were first gated for live, singlet cells based on FSC/SSC properties and 7AAD exclusion. Live singlets were then gated for CD45⁺ immune cells. Within the CD45⁺ population, macrophages were identified as CD11b⁺F4/80⁺ cells. These macrophages were further classified into M1 (CD11b⁺F4/80⁺CD11c⁺CD206⁻) and M2 (CD11b⁺F4/80⁺CD11c⁻CD206⁺) subsets. Additionally, dendritic cells were defined as CD45⁺F4/80⁻CD11c⁺ cells.


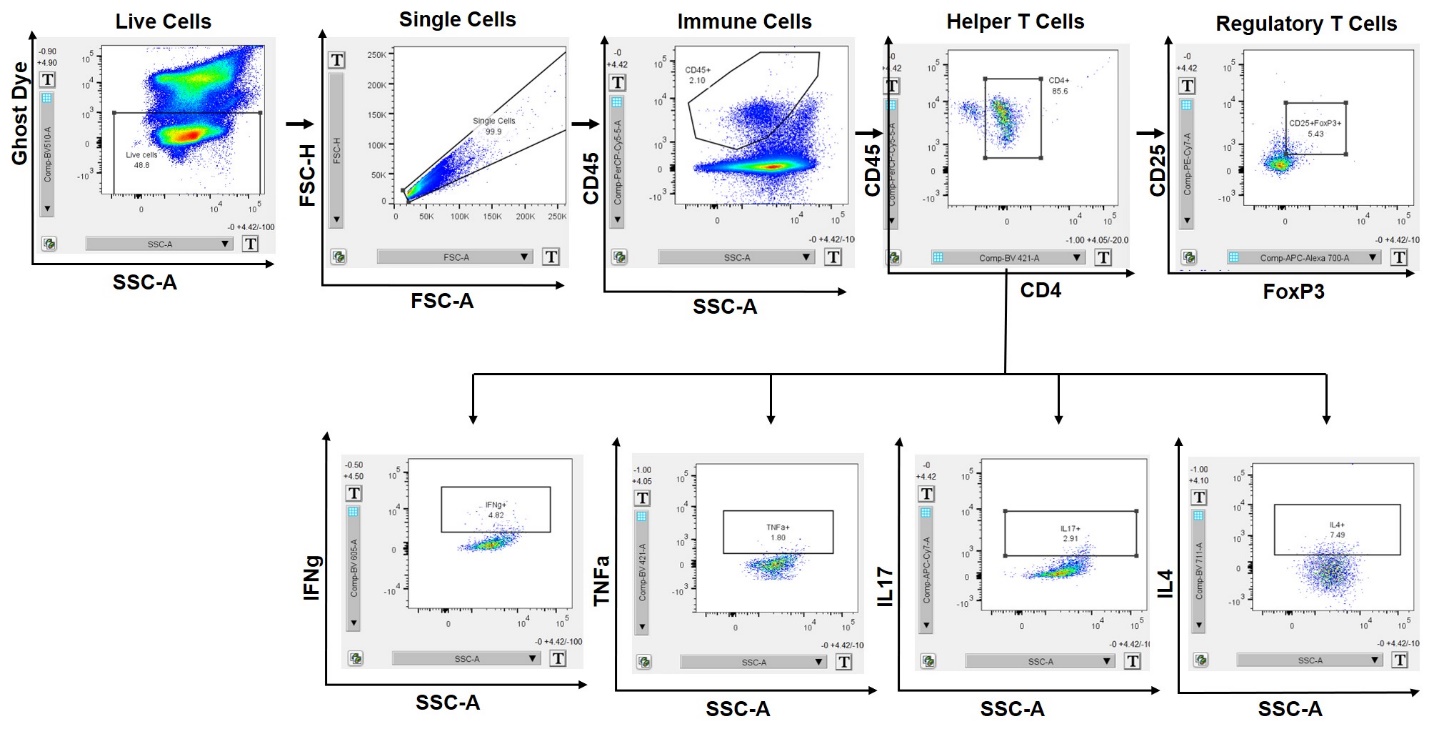


**Supplementary Figure S2.** Gating strategy for CD4-lineage T cells in uterus. Single-cell suspensions from uterine tissue were first gated to exclude dead cells and doublets, selecting for live and singlet cells. Immune cells were identified based on CD45 expression and further gated for CD4⁺ T helper cells. Regulatory T cells (Tregs) were defined as CD45⁺CD4⁺CD25⁺FoxP3⁺. Intracellular cytokine staining was then used to identify functional CD4⁺ T cell subsets expressing IFN-γ, TNF-α, IL-17, and IL-4.


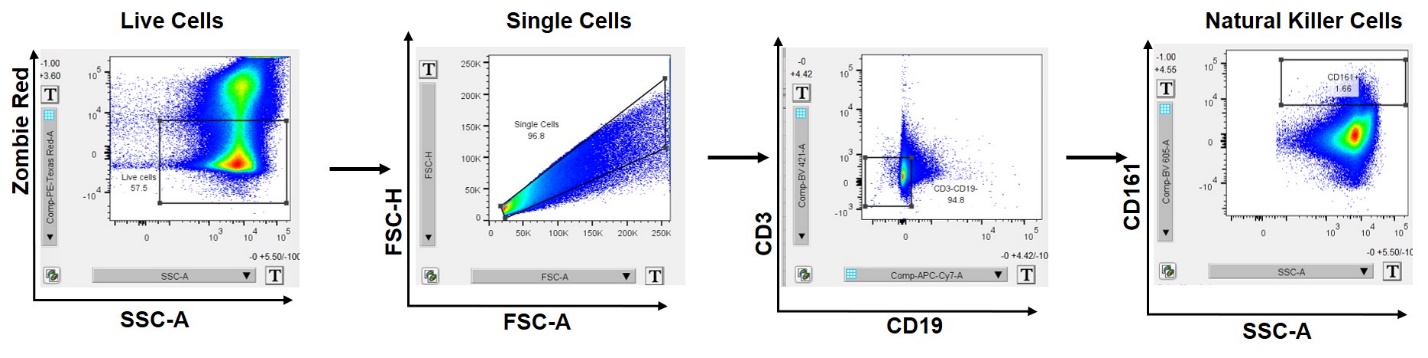


**Supplementary Figure S3.** Gating strategy for natural killer cells in uterus. Single-cell suspensions from uterine tissue were first gated to exclude dead cells using Zombie Red and to select singlet populations based on FSC-A vs FSC-H. Natural killer cells were subsequently gated as CD161⁺ within the CD3⁻CD19⁻ population.


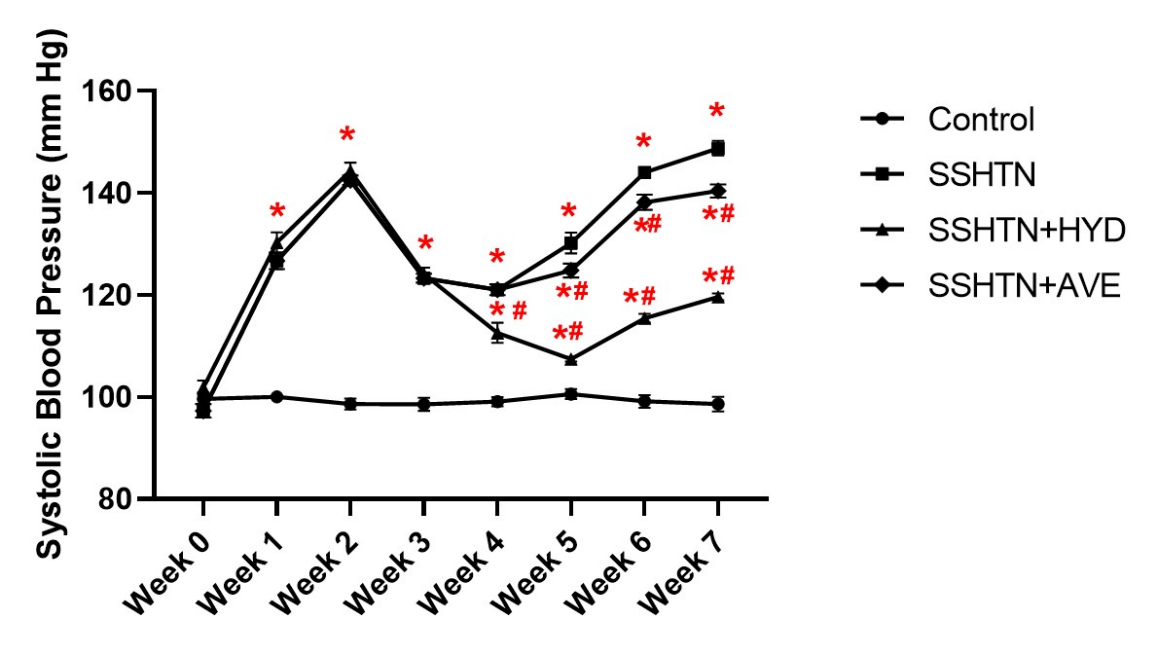


**Supplementary Figure S4:** Systolic blood pressure measures in untreated control, SSHTN, SSHTN+HYD, and SSHTN+AVE female mice. Results are presented as mean ± SEM and statistical analyses were performed with one-way ANOVAs (n=5-12 per group). *P<0.05 vs control mice and #P<0.05 vs SSHTN mice.
